# Analysis of Potential Markers of Pork Freshness Based on Volatile Organic Compounds

**DOI:** 10.3390/foods14050832

**Published:** 2025-02-28

**Authors:** Wu Wang, Yujing Wang, Peilin Weng, Yixin Zhang, Jiali Peng, Fei Ma, Hui Zhou

**Affiliations:** 1 School of Food and Biological Engineering, Hefei University of Technology, Hefei 230009, China; 2022111411@mail.hfut.edu.cn (Y.W.);; 2 Engineering Research Centre of the Ministry of Education for Agricultural Biochemicals, Hefei University of Technology, Hefei 230009, China

**Keywords:** air-stored pork, freshness, VOCs, HP-SPME-GC-MS, PLS-DA

## Abstract

Bacteria and endogenous enzymes generate volatile organic compounds (VOCs), which are posited to be the primary source of undesirable flavors in spoilt pork. Headspace solid-phase microextraction–gas chromatography–mass spectrometry (HS-SPME-GC-MS) was employed to assess the fluctuations in VOC concentrations in pork stored under tray packaging at 6–8 °C for 10 days, while total volatile basic nitrogen (TVB-N) and total viable counts (TVCs) were used to determine the quality of the pork. During storage, TVCs steadily increased, reflecting the growth of spoilage-related microorganisms, while TVB-N levels surpassed the spoilage threshold early, indicating an acceleration of the degradation process. Nine VOCs associated with pork spoilage were found by partial least squares discriminant analysis (PLS-DA), fold change (FC), and *t*-tests. The substances comprised ethyl acetate, acetoin, 3-methyl-1-butanol, 3-methylbutanal, 1-octen-3-ol, hexanal, vinyl acetate, 2-methylaziridine, and heptanal. A univariate linear regression analysis revealed a strong positive correlation (*p* < 0.001) between the gaseous total volatile basic nitrogen (G-TVBN) and the storage duration. Given that G-TVBN accurately reflects changes in pork freshness and the progression of spoilage, these results highlight the potential for dynamically monitoring the freshness and spoilage processes of pork.

## 1. Introduction

Pork is the most widely consumed meat per capita worldwide, according to data from the US Department of Agriculture’s Foreign Agricultural Service [1]. Its popularity among consumers is due to its abundance of high-quality protein and other nutrients [2,3,4]. However, microbial activity and lipid–protein oxidation make pork extremely perishable [5]. These processes can lead to changes in color and texture, slime formation, and the emergence of unpleasant odors [6,7].

At present, total volatile basic nitrogen (TVB-N) and total viable count (TVC) are extensively employed in laboratories to evaluate the freshness of pork [8,9,10]. Although these methods are highly accurate and reliable, they are time-consuming and labor-intensive, making them unsuitable for real-time monitoring [11]. In recent years, spectroscopic methods such as near-infrared (NIR) and Raman spectroscopy have become popular due to their precision and speed in assessing meat freshness [11,12,13,14,15,16]. A multivariate statistical information fusion method utilizing NIR spectroscopy has been employed to create quantitative predictive models for TVB-N and pH to evaluate pork freshness [11]. A TVC prediction algorithm utilizing hyperspectral imaging technology has been implemented for the non-destructive detection of chilled pork [17]. These non-invasive techniques provide the swift detection of chemical and biological alterations in meat products, delivering real-time and precise food safety data to regulators. Nonetheless, their intricacy and the specialized knowledge necessary for their utilization restrict their broad adoption among consumers.

Gas chromatography–mass spectrometry (GC-MS) is extensively utilized for the identification and quantification of volatile organic compounds (VOCs) [17,18,19]. The generation of off-flavors during meat deterioration is attributed to specific VOCs [20]. Consequently, GC-MS is employed to detect VOCs that indicate changes in meat freshness, offering a novel methodology for assessing pork quality. Mansur et al. utilized correlation analysis and multivariate statistics to identify signs of deterioration in beef [21]. Klein and colleagues employed thermal desorption gas chromatography–mass spectrometry (TD-GC/MS) to detect signs of deterioration in chicken breasts stored in modified environment packaging at 6 °C [22]. Additionally, Sun et al. used HS-SPME-GC-MS and correlation analysis to find possible volatile indicators of spoilage in pork [23]. Zareian et al. investigated the release of VOCs in pork stored in modified atmosphere packaging at 4 °C, although they did not focus on identifying specific spoilage indicators [24].

This study aims to quantitatively analyze the changes in VOCs during pork storage using HP-SPME-GC-MS. Specific VOCs associated with spoilage will be identified to provide a scientific basis for pork quality control. Furthermore, a dynamic indicator for quality monitoring throughout the storage process is proposed, which is expected to enable the real-time tracking and evaluation of pork quality.

## 2. Materials and Methods

### 2.1. Preparation of Pork Samples

Fresh pork was obtained from six-month-old Threeyuan pigs from a local market. Longissimus dorsi muscles, extending from the first to second lumbar vertebrae, were collected and transported to the laboratory in an icebox immediately. Subcutaneous fat and connective tissues were removed, and the meat was divided into approximately 100 g portions. These samples were then placed on trays and stored at 6–8 °C for periods of 0, 2, 3, 5, 6, 8, and 10 days. The storage experiment was conducted in a cold storage facility provided by Hefei Bingjing Refrigeration Equipment Co., Ltd. (Hefei, China). The specific equipment used includes a fan coil unit (model: FM125-900), an SM102-2 microcomputer temperature controller, and an air cooler (model: DD-2.6/15).

At each designated sampling interval, random samples were ground at high speed for one minute using a ZG-L74A grinder (Chigo, Foshan, China). Subsequently, 5.0 g of the ground meat was allocated for volatile chemical analysis using solid-phase microextraction vials, while any excess was stored in sterile bags.

### 2.2. Bacterial and Chemical Analysis

Following the Chinese National Food Safety Standard for ‘Food Microbiology Examination—Determination of Colony Count’ (GB 4789.2-2022) [25], TVC analyses were performed. In a sterile homogenizer, 10.0 g of pork was mixed with 90 mL of 0.9% sterile NaCl solution (*w*/*v*), and the mixture was then homogenized for 30 s. The overall bacterial count was used to calculate the necessary dilutions. After appropriate dilution, a 0.1 mL aliquot of the sample was evenly spread on a count agar medium-covered plate and incubated for 48 h at 37 °C. Results were expressed in logarithmic colony-forming units per gram (Log CFU/g).

According to the Chinese National Food Safety Standard ‘Determination of Volatile Basic Nitrogen in Foods’ (GB 5009.228-2016) [26], TVB-N values were determined using the automatic Kjeldahl technique. First, 50 mL of distilled water and approximately 5.0 g of pork were blended and filtered, and 10 mL of the supernatant was then mixed with 10 mL of MgO (10 g/L). Subsequently, TVB-N was quantified using an automatic Kjeldahl analyzer (Hanon, K9860, Jinan, China).

### 2.3. 16S rRNA Gene Amplicon Sequencing [27]

Dual-end sequencing of the 16S rRNA gene was performed using the Illumina NovaSeq system at Beijing Novogene Technology Co., Ltd., Beijing, China. The hypervariable V3-V4 regions of the 16S rRNA gene were amplified using primers 341F (CCTAYGGGRBGCASCAG) and 806R (GGACTACNNGGGTATCTAAT).

### 2.4. Analysis of Volatile Organic Compounds

The method developed by Chen et al. was employed to quantify the VOCs in pork [28]. VOCs were collected via solid-phase microextraction (SPME) fibers and analyzed with an Agilent 7890A gas chromatograph connected to an Agilent 7000B mass spectrometer (Agilent Technologies, Santa Clara, CA, USA). Then, 10 µL of 2-methyl-3-heptanone internal standard solution was added to the headspace vial, which was then sealed. The sample was enriched at 60 °C for 40 min, followed by adsorption for 20 min under the same conditions, using a 50/30 µm DVB/CAR/PDMS extraction fiber head (57330-U, Supelco, Bellefonte, PA, USA). Subsequently, the fiber was desorbed at 250 °C for five minutes.

Chromatographic conditions involved using an HP-5MS capillary column (30 mm × 0.25 mm × 0.25 µm) with a split ratio of 5:1, an inlet temperature of 250 °C, a flow rate of 1 mL/min, and helium as the carrier gas. Initially, the oven temperature was set to 40 °C and maintained for 2 min. It was then increased at a rate of 5 °C/min to 120 °C and held for 5 min, followed by a similar increase to 200 °C for 2 min, and finally ramped up at 8 °C/min to 250 °C, where it was maintained for 8 min. Mass spectrometry conditions were as follows: the ionization mode was EI, with an electron energy of 70 eV. The mass scan range was set from 45 to 500 *m*/*z*. The quadrupole temperature was maintained at 150 °C, the ion source temperature at 230 °C, and the GC-MS interface temperature at 250 °C.

By comparing EI mass spectra against the NIST database, VOCs with a match quality score exceeding 80% were identified. The linear retention indices (RIs) for the VOCs were calculated using standard alkane mixtures (C_5_-C_15_) from Macklin (Shanghai, China) as reference compounds [29]. The results were then validated against the NIST database (https://webbook.nist.gov/chemistry/, (accessed on 2 June 2024)) [30]. Before statistical analysis, peak regions were matched against the internal standard, reported in ng/g, to generate semi-quantitative data.

$$C(\mathrm{ng}/g)=(\frac{C_{0}\times V_{0}\times S_{1}}{S_{0}\times M})\times 1000$$

where M is the sample mass (g), C is the target volatile flavor compound concentration (ng/g), C_0_ is the internal standard concentration (μg/μL), V_0_ is the volume of the internal standard injected (mL), S_1_ is the target volatile flavor compound peak area (AU·min), and S_0_ is the internal standard peak area (AU·min).

### 2.5. Data Processing and Statistical Analysis

Due to high variability, any VOC appearing in less than 50% of the storage periods or with a detection rate below 40% at any storage time was deemed unsuitable for further statistical analysis [31]. To estimate missing values in the dataset, one-fifth of the minimum value of the relevant VOC was used [32]. Given that VOCs were identified in a semi-quantitative manner, the VOC dataset was analyzed as a quantitative continuous variable [33].

TVB-N and TVC were the main quality indicators used to assess the freshness of the pork. The samples were divided into “fresh” and “spoiled” groups according to a TVB-N threshold of less than 15 mg/100 g. T-tests were performed to evaluate significant differences in VOCs between these groups (*p* < 0.05, adjusted using the Benjamini–Hochberg process), and fold change (FC) values were computed. Principal component analysis (PCA) and partial least squares discriminant analysis (PLS-DA) were employed to evaluate the ability to categorize pork freshness according to storage length, highlighting significant VOCs using variable importance in projection (VIP) scores. The established criteria for identifying significant VOCs comprised *p* < 0.05 (BH-corrected), VIP scores ≤ 1, and FC values ≤ 0.5 or ≥2. A linear regression analysis was conducted on the overall concentration of nitrogen-containing VOCs to examine their temporal variations during storage. All data were expressed as mean ± standard deviation and utilized SPSS 20.0 and Origin 2021 software.

## 3. Results and Discussion

### 3.1. Analysis of Pork TVC and TVB-N

**Figure 1:**
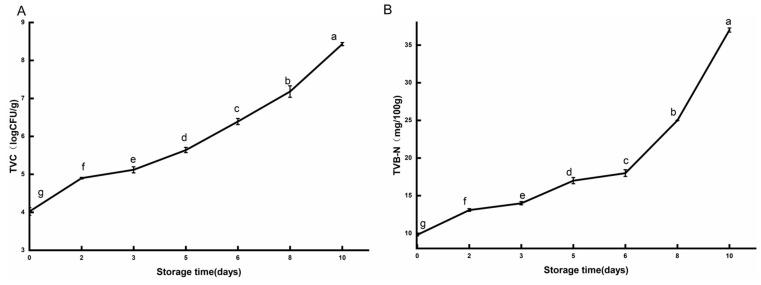
Changes in total viable count (TVC) and total volatile basic nitrogen (TVB-N) in pork over different storage periods (6–8 °C). Error bars represent standard deviation (SD). TVC value (**A**), TVB-N value (**B**). Different superscripts (a–g) indicate statistically significant differences (*p* < 0.05).

TVC and TVB-N are used as critical indicators for assessing pork freshness, with their variations throughout the storage period illustrated in Figure 1. Initially, pork exhibited a TVC value of 4.02 ± 0.1 log CFU/g, which increased steadily to 6.39 log CFU/g by day 6, exceeding the spoilage threshold of 6–7 log CFU/g for fresh meat, as reported by [34].

TVB-N acts as an indicator of protein degradation, signifying the aggregate of ammonia and other volatile amines produced by microbial activity and endogenous enzymes [35]. Initially, the TVB-N level in pork registered at 9.8 mg/100 g, rising gradually to 14 mg/100 g by day 3, just below the acceptable threshold. Initially, TVB-N concentrations exhibited a gradual increase for two primary reasons: first, microbes preferentially utilize carbohydrates as substrates in the early stages [36]; second, microbes need a period of adaptation and growth, during which their metabolic activities have not yet peaked, resulting in a slower increase in TVB-N levels. Subsequently, TVB-N content enters a rapid growth phase, showing an exponential increase in later storage stages as microbial populations and metabolic activities accelerate [35]. According to the Chinese National Food Safety Standard GB 2707-2016 (Sanitary Standard for Fresh and Frozen Livestock Meat) [37], the permissible TVB-N level in fresh meat must not exceed 15 mg/100 g.

### 3.2. 16S rRNA Gene Sequencing Analysis

**Figure 2:**
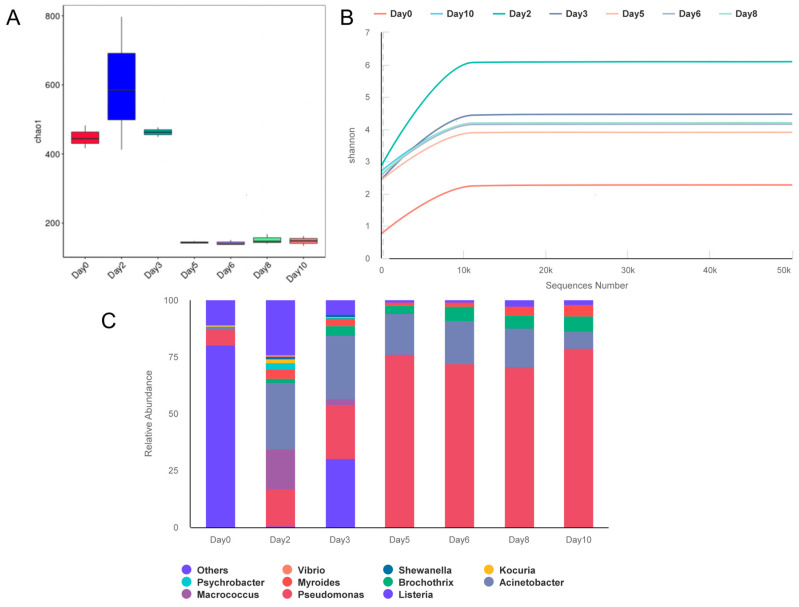
Microbial diversity and composition during pork storage: Alpha diversity index (**A**), Shannon diversity index over time (**B**), and relative abundance of dominant bacterial genera (**C**).

Sequence analysis of the 16S rRNA gene revealed that 3093 operational taxonomic units (OTUs) clustered with 97% similarity. The Chao1 index (Figure 2A), indicating high species richness, peaked on the second day of storage. Similarly, the Shannon index (Figure 2B) reached high levels, demonstrating significant evenness and diversity within the microbial community. However, these indices later showed a downward trend, suggesting a simplification in community structure and a decrease in microbial richness. All samples achieved a coverage index of 1.000, indicating that the sequencing depth was sufficient to encompass all species and ensure the completeness of the sequencing.

The composition of the microbial population underwent significant changes throughout the storage period. Despite the presence of various species, the spoiling process was dominated by a few microbial species. The ten principal bacteria depicted in Figure 2C, such as *Brochothrix, Pseudomonas, Listeria,* and *Acinetobacter,* are considered key to the degradation of air-stored pork [35,38]. Initially, *Listeria* spp. were predominant, comprising up to 80% of the total microbial population. However, during the storage period, the quantity of *Listeria* spp. progressively decreased, nearly reaching extinction by day 10. *Pseudomonas* spp., reaching a peak relative abundance of 78.7% on day 10, demonstrated a significant competitive advantage, becoming the predominant microbe in the later stages of storage.

During the mid-phase of storage, *Acinetobacter* spp. and *Macrococcus* spp. exhibited certain advantages, yet *Pseudomonas* spp. eventually surpassed them. Similarly, the relative abundance of *Brochothrix* spp. increased from the middle to late stages, peaking at 6.53% on day 6. Despite their low abundance, *Psychrobacter* spp. remained quite stable throughout the storage period.

*Pseudomonas* spp. are frequently isolated from rotten meat due to their heightened activity in aerobic environments [39,40]. Initially, these bacteria metabolize meat glucose, a precursor to VOCs such as ethanol, 3-methyl-1-butanol, and acetoin. In aerobic conditions, *Pseudomonas* spp. convert substrates into lactate and pyruvate, whereas in anaerobic conditions, they transform them into pyruvate and gluconate [41]. When nutrients are scarce, *Pseudomonas* spp. secrete extracellular proteases to degrade connective tissues between muscle fibers. This enables access to nutrients and provides a competitive edge in the microbial community [42]. Besides accelerating the spoilage process, the activity of these proteases also softens the meat and generates TVB-N [35,40]. Although *Acinetobacter* spp. are the primary spoilage bacteria in refrigerated seafood, they are not major contributors to rotting in other contexts, as they do not produce extracellular lipases, hydrogen sulfide, or trimethylamine. However, through quorum-sensing signaling molecules, *Acinetobacter* spp. can still accelerate the spoilage process by promoting the growth of other spoilage bacteria [43].

*Brochothrix thermosphacta* is commonly found as a spoilage bacterium in fresh meat stored under modified atmosphere packaging and refrigeration conditions. It is frequently observed in both low-oxygen and high-oxygen environments [44]. Ribose, glycerol, and amino acids are utilized by this bacterium as energy sources [45], resulting in the production of VOCs, such as 3-hydroxy-2-butanone, acetic acid, and alcohols, which are responsible for the generation of unpleasant odors in meat [43].

### 3.3. Analysis of VOCs by HP-SPME-GC-MS

**Figure 3:**
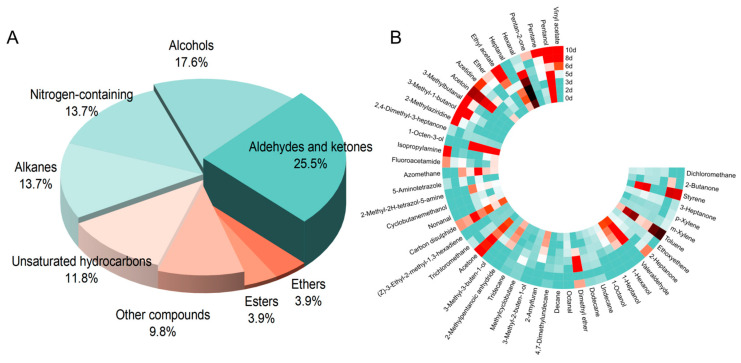
Proportions of different categories of volatile organic compounds (VOCs) in pork during storage (**A**) and a heat map of VOC concentration changes during storage (**B**). Red indicates an increase in relative content during storage, while green indicates a decrease in relative content during storage.

A total of 109 VOCs were detected using HP-SPME-GC-MS. After excluding data with high variability, 51 VOCs were retained for further analysis (Table S1). Among these, there were 7 (cyclo)alkanes, 14 aldehydes and ketones, 2 esters, 2 alcohols, 6 unsaturated hydrocarbons, 2 ethers, 7 nitrogen-containing compounds, and 5 other types of compounds. Figure 3A,B depict the proportional composition and concentration changes of VOCs during pork storage.

Aldehydes are regarded as the primary volatile compounds formed during the oxidative degradation of meat. These compounds are predominantly produced via the hydrolysis of triglycerides and the metabolic pathways of fatty acids, and they can also be generated through the transamination of amino acids. Common aldehydes detected in meat include hexanal, nonanal, heptanal, and 3-methylbutanal. Among these, hexanal is considered a key contributor to meat flavor development [46]. In agreement with the findings of Song et al. [47], the concentration of hexanal was initially high at the beginning of storage but then decreased significantly over time. In the later stages of storage, the degradation of pork proteins becomes closely associated with a rapid increase in the synthesis of 3-methylbutanal, which can be detected as early as day 5 [48]. This suggests that 3-methylbutanal could serve as a potential marker for identifying non-fresh pork [23].

Ketones are recognized as byproducts of the Strecker degradation [49]. Among all ketones detected, acetoin exhibited a significant change in concentration during the storage period, increasing from 29.61 ng/g on day 5 to 168.03 ng/g. Acetoin can be formed through the oxidation of 2,3-butanediol, enzymatic decarboxylation of 2-acetolactate, or as a byproduct of glucose metabolism and microbial degradation [41,47]. Numerous studies have proposed acetoin as a reliable indicator of spoilage in stored chicken breast and pork [22,23,50,51,52].

Lipoxygenase and peroxidase convert linoleic acid in muscle into alcohols, which are believed to contribute to the volatile flavors of the meat [46]. During storage, the concentration of 3-methylbutanal increases, whereas the levels of known alcohols, such as 1-octen-3-ol, heptanol, and pentanol, decrease. Studies have demonstrated that 1-hexanol and 1-octanol, volatile compounds characteristic of fresh pork, are entirely absent in spoiled meat [47]. Notably, 1-octen-3-ol, which imparts a distinct mushroom-like aroma and plays a critical role in enhancing pork’s flavor, is predominantly present during the early storage stages. This compound is formed via the enzymatic breakdown of eicosapentaenoic acid by 15-lipoxygenase and arachidonic acid by 12-lipoxygenase [53]. However, its concentration markedly decreases by day 5 of storage [30,54]. Wen et al. suggested that pentanol and 1-octen-3-ol could serve as potential spoilage markers for lamb [51]. Similarly, Mikš-Krajnik et al. identified 3-methylbutanal as a promising volatile spoilage marker for chicken breast [55], while Huang and Xie proposed that both 3-methylbutanal and 1-octen-3-ol could serve as reliable indicators of spoilage in grouper [56].

### 3.4. Selection of Indicators for Pork Freshness

**Figure 4:**
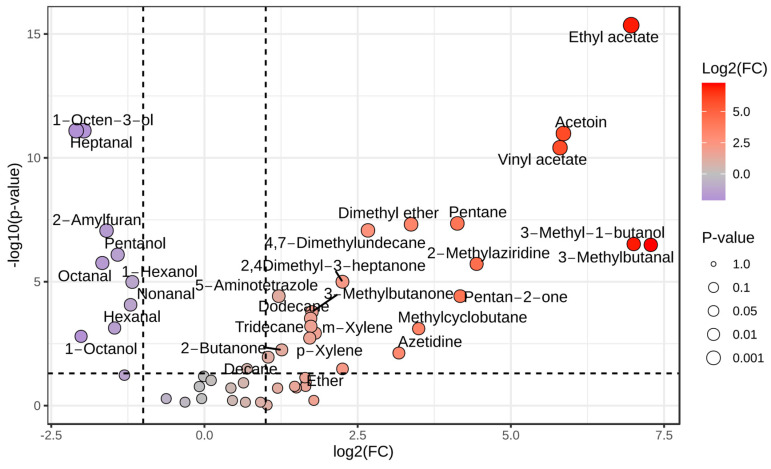
Differentially expressed compounds during pork storage (|log_2_FC| > 2, *p* < 0.05). The circles with darker colors represent the compounds with the most significant VOC concentration differences between fresh and spoiled pork.

By applying the Benjamini–Hochberg method to adjust for multiple comparisons (*p* < 0.05), the *t*-test identified 32 VOCs with significant differences between ‘fresh’ and ‘spoiled’ pork samples. FC analysis revealed 30 compounds with FC values above 2, indicating an increase, and 10 compounds with FC values less than 0.5, indicating a decrease. These results prompted the creation of a volcano plot (Figure 4), which illustrates the statistically significant compounds and the magnitude of their expression changes. Integrating these methodologies enabled the identification of 21 VOCs that were significantly elevated due to spoilage, while 10 were found to notably decline (*p* < 0.05, BH-corrected).

**Figure 5:**
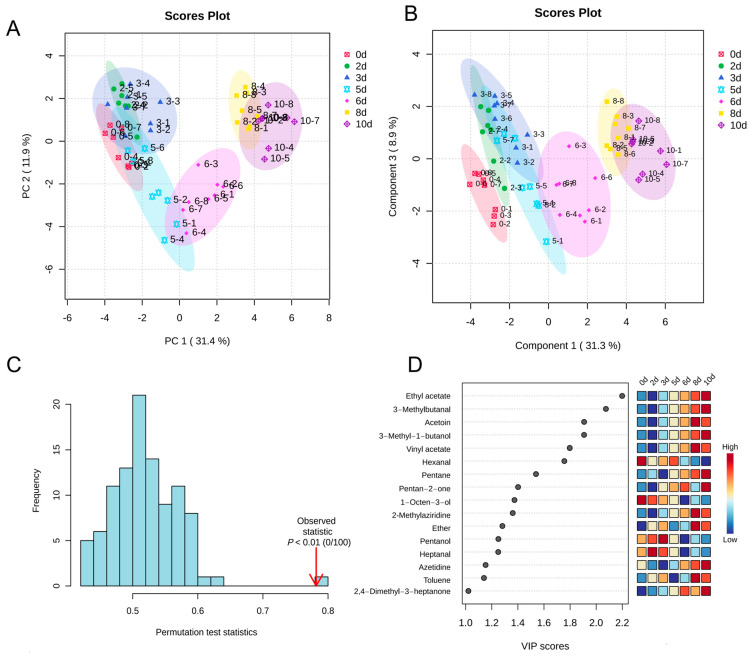
Principal component analysis (PCA) and significant differential metabolite analysis of volatile compounds in fresh and spoiled pork. PCA scores plot (**A**), partial least squares discriminant analysis (PLS-DA) scores plot (**B**), permutation test with 100 iterations (**C**), and variable importance in projection scores (VIP > 1) (**D**).

According to the PCA results displayed in Figure 5A, PC1 explains 31.4% of the total variance, whereas PC2 explains 11.9%. This analysis clarifies the pattern of sample changes over time and highlights the significant impact of storage duration on VOC characteristics. Fresh pork samples are located in the negative quadrant of PC1 and exhibit a gradual transition towards the positive quadrant with increasing storage time. The considerable overlap observed for the results from day 2 and day 3 indicates that their VOC profiles are highly similar. Based on this foundation, variables that significantly contribute to distinguishing pork samples by storage duration were further extracted using PLS-DA. The PLS-DA score plot in Figure 5B illustrates the distinction between samples. However, the classification ability of PLS-DA was found to be limited, likely due to the high similarity of VOC components in fresh pork samples. In the early stages of storage, fresh samples are minimally influenced by microbial metabolism and enzymatic activities, resulting in negligible changes in the types and concentrations of VOCs. This high degree of consistency within the group complicates the ability of PLS-DA to extract clear classification features, thus diminishing its effectiveness in differentiating between groups. Conversely, samples stored for longer periods are characterized by more marked differences, enhancing the model’s classification capabilities. The model fitting metrics, R^2^ and Q^2^, were 0.96 and 0.90, respectively, indicating strong predictive performance, as an R^2^ value greater than 0.7 typically signifies strong predictive capability. Cross-validation confirmed the stability and reliability of the PLS-DA model (Figure 5C). Sixteen VOCs with scores exceeding one were identified by the analysis of VIP scores. The dynamic patterns of these VOCs are clearly illustrated by the VIP score heatmap, with significant concentration fluctuations over various storage periods being demonstrated (Figure 5D).

In this study, 15 VOCs were initially identified through univariate and multivariate analysis methods (Table 1). Six VOCs—2,4-dimethyl-3-heptanone, azetidine, ether, pentan-2-one, pentane, and pentanol—were excluded as potential markers due to their lack of a consistent correlation with freshness or storage time. Among the remaining nine VOCs, most were observed to appear or disappear by day 5 or day 6 of storage. This behavior may be attributed to protein degradation and lipid oxidation, where initial concentrations of these VOCs remain below the detection threshold and gradually increase to detectable levels. Conversely, some VOCs may diminish due to chemical reactions or metabolic activities. While these VOCs can serve as indicators of spoilage, they are limited in providing precise timing information regarding the spoilage process. Notably, hexanal was found to lack a reliable freshness signal due to its inability to induce a visible color change, thereby restricting its applicability in rapid meat freshness assessment.

**Figure 6:**
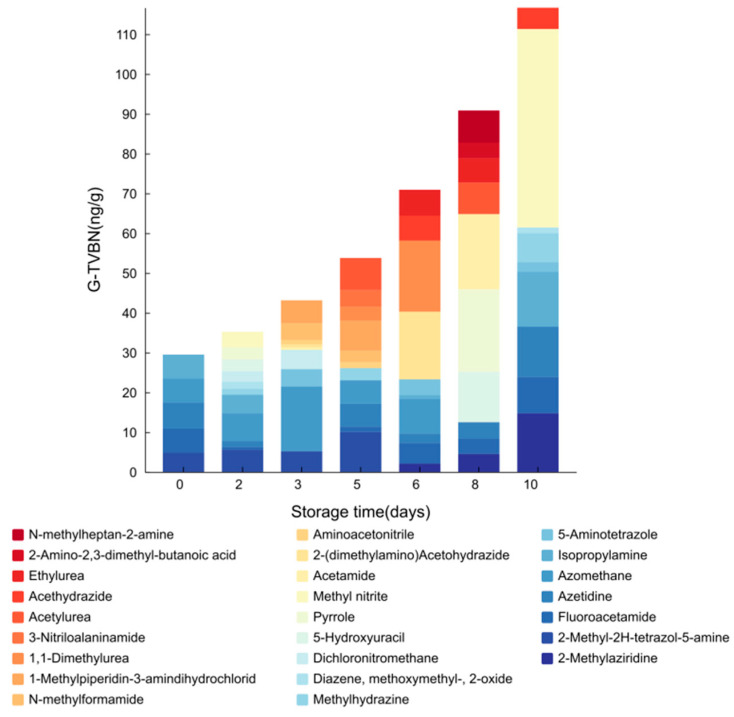
Temporal variation in the cumulative daily concentration of gaseous total volatile basic nitrogen (G-TVBN) during pork storage.

Given the limitations of individual VOCs in capturing the dynamic changes in pork freshness over time, a comprehensive index known as gaseous total volatile basic nitrogen (G-TVBN) was developed. This index is derived from the cumulative concentrations of all nitrogen-containing VOCs. A univariate linear regression analysis demonstrated a highly significant correlation between the duration of storage and the G-TVBN values (*p* < 0.001), with the model explaining 97.6% of the variability in G-TVBN concentrations, evidenced by an R^2^ value of 0.976. The regression formula, Y = 19.5 + 8.91x, highlights the substantial impact of storage duration on the concentrations of G-TVBN, underscoring its potential as a reliable indicator for monitoring meat spoilage. Figure 6 visually represents the variations in G-TVBN concentrations throughout the storage period, illustrating the cumulative levels and providing a graphical representation of the observed trends.

## 4. Conclusions

This study systematically investigated the spoilage process of pork stored at 6–8 °C through microbial community analysis and VOC profiling. The results showed that days 3–5 of storage represent a critical period of quality deterioration, accompanied by significant proliferation of spoilage-related bacteria, such as *Pseudomonas* spp. and *Listeria* spp., as well as characteristic changes in VOCs. Hexanal was identified as a stable spoilage biomarker consistently present throughout the storage period, confirming its potential as a reliable indicator of meat spoilage. Meanwhile, changes in compounds such as acetoin, 3-methyl-1-butanol, 3-methylbutanal, 1-octen-3-ol, vinyl acetate, 2-methylaziridine, and heptanal suggest their potential as markers for pork spoilage. A strong correlation was observed between the G-TVBN index and storage time (R^2^ = 0.976), providing an effective tool for the real-time monitoring of pork freshness.

This study established a dual monitoring system combining VOCs and the G-TVBN index, offering a novel approach to the real-time tracking of meat spoilage. These findings lay a solid theoretical foundation for the development of intelligent packaging systems based on VOC sensing technologies, with significant implications for enhancing the quality control systems of the meat supply chain.

## Figures and Tables

**Table 1:** Fifteen candidate volatile organic compounds (VOCs) associated with pork spoilage and identified based on univariate analysis (*t*-test, *p* < 0.05; FC > 2 or FC < 0.5) and multivariate analysis (PLS-DA, VIP > 1.0).

VOC	Storage Time (Day)							log_2_(FC)	*p*. Adjusted	VIP
	0	2	3	5	6	8	10			
1-Octen-3-ol	44.82 ± 13.61 ^a^	43.36 ± 5.07 ^a^	36.93 ± 9.08 ^a^	19.11 ± 6.13 ^b^	ND	ND	ND	0.23	*p* < 0.05	1.37
2,4-Dimethyl-3-heptanone	1.4 ± 0.49 ^a^	1.56 ± 0.54 ^a^	1.88 ± 0.23 ^a^	1.89 ± 1.1 ^a^	2.02 ± 0.33 ^a^	ND	2.27 ± 0.36 ^a^	4.76	*p* < 0.05	1.02
2-Methylaziridine	ND	ND	ND	ND	2.1 ± 0.17 ^c^	4.51 ± 1.68 ^b^	14.73 ± 1.77 ^a^	21.72	*p* < 0.05	1.36
3-Methyl-1-butanol	ND	ND	ND	2.14 ± 0.72 ^c^	4.34 ± 0.01 ^c^	16.58 ± 4.93 ^b^	50.76 ± 5.56 ^a^	128.38	*p* < 0.05	1.91
3-Methylbutanal	ND	ND	ND	ND	0.66 ± 0.29 ^c^	5.8 ± 2.25 ^b^	19.34 ± 4.97 ^a^	155.69	*p* < 0.05	2.07
Acetoin	ND	ND	ND	29.62 ± 4.32 ^d^	93.55 ± 3.91 ^c^	169.93 ± 17.47 ^b^	192.04 ± 19.57 ^a^	57.98	*p* < 0.05	1.91
Azetidine	6.59 ± 2.59 ^b^	1.48 ± 0.19 ^cd^	ND	5.8 ± 0.19 ^b^	2.35 ± 0.66 ^cd^	4.09 ± 0.38 ^bc^	12.66 ± 3.73 ^a^	9.02	*p* < 0.05	1.15
Ether	ND	2.74 ± 0.98 ^c^	3.68 ± 0.92 ^bc^	ND	ND	5.07 ± 0.82 ^ab^	6.8 ± 2.85 ^a^	4.77	*p* < 0.05	1.28
Ethyl acetate	ND	ND	ND	4.41 ± 1.42 ^d^	10.4 ± 2.48 ^c^	30.84 ± 5.02 ^b^	63.14 ± 6.63 ^a^	124.75	*p* < 0.05	2.20
Heptanal	7.47 ± 2.68 ^b^	11.68 ± 2.34 ^a^	10.36 ± 3.1 ^ab^	4.34 ± 1.02 ^c^	ND	ND	ND	0.26	*p* < 0.05	1.25
Hexanal	309.16 ± 40.13 ^b^	405.51 ± 15.87 ^a^	406.81 ± 26.97 ^a^	267.47 ± 43.84 ^c^	25.37 ± 10.7 ^d^	3.64 ± 0.36 ^d^	2.7 ± 0.42 ^d^	0.36	*p* < 0.05	1.76
Pentan-2-one	ND	ND	0.82 ± 0.2 ^bc^	0.78 ± 0.29 ^c^	1.24 ± 0.27 ^b^	ND	7.32 ± 0.58 ^a^	18.04	*p* < 0.05	1.40
Pentane	2.38 ± 0.17 ^c^	2.89 ± 0.22 ^c^	ND	4.77 ± 0.09 ^b^	5 ± 1.34 ^b^	5.39 ± 1 ^b^	17.01 ± 2.14 ^a^	17.47	*p* < 0.05	1.54
Pentanol	54.62 ± 6.41 ^b^	59.33 ± 5.73 ^ab^	64.46 ± 6.27 ^a^	34.95 ± 2.95 ^c^	6.47 ± 0.14 ^e^	16.67 ± 4 ^d^	17.79 ± 0.56 ^d^	0.37	*p* < 0.05	1.25
Vinyl acetate	ND	ND	ND	4.06 ± 1.23 ^c^	11.58 ± 1.93 ^b^	18.14 ± 4.18 ^a^	16.18 ± 3.9 ^a^	55.79	*p* < 0.05	1.80

Means with different superscripts in the same row are significantly different (*p*  <  0.05), and ND indicates not detected.

## Data Availability

The original contributions presented in the study are included in the article/Supplementary Materials, further inquiries can be directed to the corresponding author.

## Supplementary Materials

The following supporting information can be downloaded at: https://www.mdpi.com/article/10.3390/foods14050832/s1, Table S1: Changes in the concentrations of VOCs during pork storage.
