# Supplementary material for: Analysis of Potential Markers of Pork Freshness Based on Volatile Organic Compounds
Source: Foods. 2025 Feb 28;14(5):832. doi: 10.3390/foods14050832 (PMC11898832; doi:10.3390/foods14050832)
Supplement: Supplementary file 1 [file foods-14-00832-s001.zip › foods-3462903-supplementary.pdf]

Analysis of Potential Markers of Pork Freshness Based on Volatile Organic Compounds

Wu Wang [1,2,*], Yujing Wang [1], Peilin Weng [1], Yixin Zhang [1], Jiali Peng [1], Fei Ma [1,2] and Hui Zhou [1,2]

[1] School of Food and Biological Engineering, Hefei University of Technology, Hefei 230009, China
[2] Engineering Research Centre of the Ministry of Education for Agricultural Biochemicals, Hefei University of Technology, Hefei 230009, China
* Correspondence: 1994800074@hfut.edu.cn

Table S1. Changes in the concentrations of VOCs during pork storage (mean ± standard deviation).

RT (min)	CAS	Compounds	Storage time(days) 0	2	3	5	6	8	10	Calculated RI	Reference RI	Identification
**Alkanes**												
16.87	17301-32-5	4,7-Dimethylundecane	ND	ND	ND	0.81 ± 0.02^{a}	1.31 ± 1.15^{a}	ND	0.78 ± 0.03^{a}	1066.12	-	-
5.33	109-66-0	Pentane	2.38 ± 0.17^{c}	2.89 ± 0.22^{c}	ND	4.77 ± 0.09^{b}	5 ± 1.34^{b}	5.39 ± 1^{b}	17.01 ± 2.14^{a}	500	500[1]	MS
17.99	1120-21-4	Undecane	2.64 ± 1.13^{a}	2.08 ± 0.63^{a}	2.45 ± 0.39^{a}	2.63 ± 0.83^{a}	2.27 ± 0.95^{a}	ND	1.77 ± 0.5a	1100	1100	MS
21.31	112-40-3	Dodecane	1.71 ± 0.61^{ab}	ND	1.47 ± 0.52^{b}	2.46 ± 0.87^{a}	1.99 ± 0.51^{ab}	ND	1.43 ± 0.77^{b}	1200	1200	MS
15.17	124-18-5	Decane	1.35 ± 0.43^{a}	ND	1.31 ± 0.08^{a}	1.45 ± 0.48^{a}	2.01 ± 1.5^{a}	ND	ND	1000	1000	MS
25.68	629-50-5	Tridecane	0.63 ± 0.25^{a}	0.45 ± 0.15^{ab}	ND	0.55 ± 0.19^{ab}	0.48 ± 0.21^{ab}	0.97 ± 0.01^{ab}	0.58 ± 0.07^{a}	1300	1300	MS
10.06	598-61-8	Methylcyclobutane	0.58 ± 0.26^{c}	ND	0.62 ± 0.53^{c}	2.6 ± 0.33^{b}	4.85 ± 1.93^{a}	1.08 ± 0.27^{c}	1.1 ± 0.33^{c}	828.07	-	-
**Aldehydes and Ketones**												
11.9	106-35-4	3-Heptanone	3.44 ± 0.66^{ab}	3.26 ± 0.65^{ab}	4.26 ± 1.66^{a}	3.5 ± 0.69^{ab}	3.3 ± 0.4^{ab}	ND	2.53 ± 0.06^{b}	892.98	887	RI
6.7	107-87-9	Pentan-2-one	ND	ND	0.82 ± 0.2b^{c}	0.78 ± 0.29^{c}	1.24 ± 0.27^{b}	ND	7.32 ± 0.58^{a}	695	686	RI
11.93	110-43-0	2-Heptanone	4.16 ± 1.66^{b}	4.36 ± 1.72^{b}	3.72 ± 1.03^{b}	2.62 ± 0.03^{b}	ND	3.81 ± 1.19^{b}	9.81 ± 2.88^{a}	894.06	889	RI
14.5	18641-71-9	2,4-Dimethyl-3-heptanone	1.4 ± 0.49^{a}	1.56 ± 0.54^{a}	1.88 ± 0.23^{a}	1.89 ± 1.1^{a}	2.02 ± 0.33^{a}	ND	2.27 ± 0.36^{a}	975.57	-	-
7.14	513-86-0	Acetoin	ND	ND	ND	29.62 ± 4.32^{d}	93.55 ± 3.91^{c}	169.93 ± 17.47^{b}	192.04 ± 19.57^{a}	714.95	720	RI
4.41	67-64-1	Acetone	ND	11.91 ± 2.29^{b}	12.91 ± 7.71^{b}	10.19 ± 3.56^{b}	16.26 ± 0.21^{b}	13.45 ± 3.99^{b}	27.16 ± 6.7^{a}	-	-	-
5.5	78-93-3	2-Butanone	1.74 ± 0.51^{a}	ND	2.53 ± 0.14^{a}	2.48 ± 0.77^{a}	ND	6.84 ± 1.01^{a}	ND	600	602	RI
6.84	110-62-3	Valeraldehyde	12.51 ± 2.34^{c}	28.91 ± 2.45^{a}	22.88 ± 3.54^{b}	20.88 ± 8.34^{b}	ND	ND	ND	703.15	698	RI
12.25	111-71-7	Heptanal	7.47 ± 2.68^{b}	11.68 ± 2.34^{a}	10.36 ± 3.1^{ab}	4.34 ± 1.02^{c}	ND	ND	ND	904.57	901	RI
15.21	124-13-0	Octanal	3.2 ± 0.97^{a}	3.78 ± 1.42^{a}	3.43 ± 0.9^{a}	1.72 ± 0.62^{b}	ND	ND	ND	1001.05	1004	RI
18.19	124-19-6	Nonanal	4.1 ± 1.79^{ab}	5.73 ± 1.19^{a}	5.07 ± 1.6^{a}	2.95 ± 0.66^{b}	ND	ND	ND	1105.92	1102	RI
6.14	590-86-3	3-Methylbutanal	ND	ND	ND	ND	0.66 ± 0.29^{c}	5.8 ± 2.25^{b}	19.34 ± 4.97^{a}	650.54	649	RI
9.33	66-25-1	Hexanal	309.16 ± 40.13^{b}	405.51 ± 15.87^{a}	406.81 ± 26.97^{a}	267.47 ± 43.84^{c}	25.37 ± 10.7^{d}	3.64 ± 0.36^{d}	2.7 ± 0.42^{d}	802.9	800	RI
**Esters**												
5.24	108-05-4	Vinyl acetate	ND	ND	ND	4.06 ± 1.23^{c}	11.58 ± 1.93^{b}	18.14 ± 4.18^{a}	16.18 ± 3.9^{a}	-	-	MS
5.55	141-78-6	Ethyl acetate	ND	ND	ND	4.41 ± 1.42^{d}	10.4 ± 2.48^{c}	30.84 ± 5.02^{b}	63.14 ± 6.63^{a}	622.14	612	RI
**Alcohols**												
11.27	111-27-3	1-Hexanol	11.93 ± 2.79^{a}	10.12 ± 4.18^{ab}	8.79 ± 3.41^{ab}	7.02 ± 2.64^{b}	ND	ND	ND	870.64	867	RI
14.31	111-70-6	1-Heptanol	4.09 ± 1.47^{b}	5.44 ± 0.71^{a}	4.75 ± 0.75^{ab}	1.89 ± 0.33^{c}	ND	ND	ND	971.53	969	RI
17.16	111-87-5	1-Octanol	3.33 ± 1.15^{a}	2.62 ± 0.9^{a}	2.35 ± 0.73^{a}	0.8 ± 0.23^{b}	ND	ND	ND	1070.26	1068	RI

7.68	123-51-3	3-Methyl-1-butanol	ND	ND	ND	2.14 ± 0.72^{c}	4.34 ± 0.01^{c}	16.58 ± 4.93^{b}	50.76 ± 5.56^{a}	737.1	730	RI
14.53	3391-86-4	1-Octen-3-ol	44.82 ± 13.61^{a}	43.36 ± 5.07^{a}	36.93 ± 9.08^{a}	19.11 ± 6.13^{b}	ND	ND	ND	978.62	986	RI
6.55	4415-82-1	Cyclobutanemethanol	4.33 ± 1.23^{a}	ND	3.65 ± 1.13^{ab}	2.95 ± 0.26^{b}	ND	ND	ND	691.65	-	MS
8.72	556-82-1	3-Methyl-2-buten-1-ol	1.98 ± 0.79^{a}	1.68 ± 0.35^{ab}	1.48 ± 0.38^{ab}	1.4 ± 0.34^{ab}	1.15 ± 0.33^{b}	ND	ND	779.02	778	RI
8.47	71-41-0	Pentanol	54.62 ± 6.41^{b}	59.33 ± 5.73^{ab}	64.46 ± 6.27^{a}	34.95 ± 2.95^{c}	6.47 ± 0.14^{e}	16.67 ± 4^{d}	17.79 ± 0.56^{d}	768.83	779	RI
7.61	763-32-6	3-Methyl-3-buten-1-ol	1.13 ± 0.49^{a}	1.05 ± 0.27^{a}	1.4 ± 0.31^{a}	3.76 ± 1.13^{a}	ND	ND	ND	733.89	730	RI
**Unsaturated hydrocarbons**												
11.11	100-41-4	Ether	ND	2.74 ± 0.98^{c}	3.68 ± 0.92^{bc}	ND	ND	5.07 ± 0.82^{ab}	6.8 ± 2.85^{a}	865.3	868	RI
8.55	108-88-3	Toluene	7.29 ± 2.7^{d}	164.87 ± 47.45^{c}	232.4 ± 41.97^{bc}	6.48 ± 2.07^{d}	7.14 ± 3.22^{d}	286.58 ± 105.22^{a}	335.78 ± 72.74^{a}	772	773	RI
11.97	100-42-5	Styrene	ND	56.74 ± 18.98^{c}	88.01 ± 23.78^{bc}	ND	ND	121.69 ± 39.71^{a}	109.36 ± 16.83^{ab}	895.18	890	RI
11.77	106-42-3	p-Xylene	ND	1.54 ± 0.27^{b}	1.59 ± 0.49^{b}	1.81 ± 0.2^{b}	1.74 ± 0.04^{b}	ND	3.25 ± 1^{a}	888.29	878	RI
11.42	108-38-3	m-Xylene	1.75 ± 0.53^{b}	2.02 ± 0.84^{b}	2.13 ± 0.72^{b}	2.06 ± 0.35^{b}	1.66 ± 0.62^{b}	2.05 ± 0.77^{b}	3.75 ± 1.14^{a}	876.01	861	RI
16.17	74752-97-9	(Z)-3-Ethyl-2-methyl-1,3-hexadiene	0.92 ± 0.03^{b}	0.88 ± 0.15^{b}	1.07 ± 0.04^{a}	ND	ND	ND	ND	-	-	-
**Ethers**												
5.35	109-92-2	Ethoxyethene	2.44 ± 0.2^{a}	3.33 ± 1.21^{a}	2.71 ± 0.75^{a}	2.46 ± 0.38^{a}	2.5 ± 0.31^{a}	3.5 ± 0.83^{a}	ND	524.69	-	MS
4.26	115-10-6	Dimethyl ether	ND	ND	6.14 ± 1.6^{b}	19.94 ± 2.58^{a}	20.7 ± 3.76^{a}	ND	8.35 ± 2.39^{b}	-	-	-
**Nitrogen-containing**												
5.5	503-29-7	Azetidine	6.59 ± 2.59^{b}	1.48 ± 0.19^{cd}	ND	5.8 ± 0.19^{b}	2.35 ± 0.66^{cd}	4.09 ± 0.38^{bc}	12.66 ± 3.73^{a}	977.7	-	MS
7.72	75-55-8	2-Methylaziridine	ND	ND	ND	ND	2.1 ± 0.17^{c}	4.51 ± 1.68^{b}	14.73 ± 1.77^{a}	738.56	-	-
11.81	4418-61-5	5-Aminotetrazole	ND	ND	4.33 ± 0.29^{a}	ND	3.97 ± 0.08^{a}	ND	2.42 ± 0.98^{b}	515.61	-	MS
14.66	6154-04-7	2-Methyl-2H-tetrazol-5-amine	4.81 ± 0.18^{b}	5.59 ± 0.9^{3b}	5.19 ± 0.48^{b}	10.02 ± 1.12^{a}	ND	ND	ND	738.57	-	MS
11.49	75-31-0	Isopropylamine	6.02 ± 2.21^{b}	4.66 ± 1.21^{b}	ND	ND	0.92 ± 0.28^{c}	ND	14.29 ± 0.77^{a}	889.85	-	MS
7.87	640-19-7	Fluoroacetamide	6.09 ± 1.04^{b}	0.7 ± 0.32^{d}	ND	1.33 ± 0.36^{d}	5.17 ± 1.47^{bc}	3.88 ± 0.05^{c}	9.12 ± 2.58^{a}	983.03	-	-
4.44	503-28-6	Azomethane	5.98 ± 1.23^{c}	6.97 ± 0.03^{bc}	16.29 ± 1.78^{a}	5.91 ± 2.07^{c}	8.77 ± 1.1^{b}	ND	ND	-	-	-
**Other compounds**												
5.61	67-66-3	Trichloromethane	1.17 ± 0.31^{b}	1.23 ± 0.35^{b}	1.07 ± 0.31^{b}	2.59 ± 0.48^{a}	2.3 ± 0.82^{a}	ND	2.83 ± 0.71^{a}	608.01	615	RI
4.65	75-09-2	Dichloromethane	3.16 ± 0.87^{a}	3.46 ± 0.43^{a}	2.74 ± 0.69^{a}	2.59 ± 1.13^{a}	2.89 ± 1.29^{a}	ND	ND	-	531	MS
14.94	3777-69-3	2-Amylfuran	8.99 ± 1.88^{a}	8.59 ± 3.49^{a}	6.27 ± 1.81^{ab}	5.09 ± 2.22^{b}	ND	ND	ND	992.08	996	-
4.74	75-15-0	Carbon disulphide	11.42 ± 2.46^{a}	6.32 ± 2.5^{c}	12.14 ± 4.72^{b}	9.69 ± 3.16^{bc}	30.01 ± 1.62^{a}	8.23 ± 2.72^{bc}	9.02 ± 1.31^{bc}	-	536	MS

14.5	63169-61-9	2-Methylpentanoic anhydride	7.81 ± 4.48^{a}	9.81 ± 3.8^{a}	8.82 ± 4.48^{a}	7.25 ± 2.99^{a}	ND	ND	ND	992.09	-	-

Reference RI values were obtained from the NIST Chemistry WebBook (https://webbook.nist.gov/chemistry/).

Means with different superscripts in same row are significantly different ($P < 0.05$),and ND indicates not detected.

Column Descriptions:

RT (min): Retention time (minutes).

CAS: Chemical Abstracts Service (CAS) registry number of the compound.

Calculated RI: Experimentally determined retention index.

Reference RI: Literature-reported retention index.

Identification: Method of compound identification (MS: mass spectrometry; RI: retention index matching).
